# Film-Shaped Self-Powered Electro-Osmotic Micropump Array

**DOI:** 10.3390/mi14040785

**Published:** 2023-03-31

**Authors:** Toshiro Yamanaka, Fumihito Arai

**Affiliations:** Department of Mechanical Engineering, School of Engineering, The University of Tokyo, Tokyo 113-8656, Japan

**Keywords:** micropump array, biofuel cell, electro-osmotic flow, glucose, perfusion

## Abstract

This paper reports a new concept of a film-shaped micropump array for biomedical perfusion. The detailed concept, design, fabrication process, and performance evaluation using prototypes are described. In this micropump array, an open circuit potential (OCP) is generated by a planar biofuel cell (BFC), which in turn generates electro-osmotic flows (EOFs) in multiple through-holes arranged perpendicular to the micropump plane. The micropump array is thin and wireless, so it can be cut like postage stamps, easily installed in any small location, and can act as a planar micropump in solutions containing the biofuels glucose and oxygen. Perfusion at local sites are difficult with conventional techniques using multiple separate components such as micropumps and energy sources. This micropump array is expected to be applied to the perfusion of biological fluids in small locations near or inside cultured cells, cultured tissues, living organisms, and so on.

## 1. Introduction

Recent advances in cell biology and tissue engineering have increased the demand for cell culture. In addition, a new field of bio-actuators [1] is emerging, which uses living tissues, such as muscles, as mechanical actuators. In order to maintain and culture living cells, substances such as oxygen and nutrients must be continuously supplied by biological fluids. Therefore, artificial fluid perfusion mechanisms are needed to replace the heart and vascular system. Small, wireless pumps are desirable to perform perfusion at localized sites in and outside the body. Furthermore, the size of such micropumps should be adjustable to fit to the cells, tissues, and organs.

**Figure 1:**
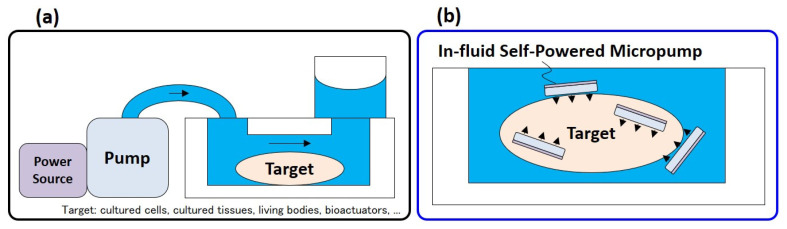
Perfusion methods. (**a**) Conventional perfusion with an external pump. (**b**) Perfusion via in-fluid self-powered micropumps.

There are various types of pumping principles for perfusion, including gravity [2,3], centrifugation [4,5], syringes [6], vacuum [7,8], peristalsis [9,10,11], surface tension [12,13], electrokinetic mechanisms [14,15], osmosis [16,17], electro-osmosis [18,19], and so on. As shown in Figure 1a, conventional perfusion techniques are generally configured with the pump and energy source located outside the chamber. In such a configuration, perfusion at a local site is difficult.

Electro-osmotic flow (EOF) is a suitable mechanism for localized perfusion because it occurs prominently on the microscale. Many microfluidic devices for perfusion that integrate a micropump and a flow path based on MEMS technology [20] have been proposed. However, the closed flow path and chamber limit the volume that can accommodate living cells as shown in Figure 1a. Microfluidic devices tend to be flat due to MEMS fabrication techniques, so it is generally not easy to accommodate larger cell samples such as spheroids or organoids in the devices. Additionally, enlarging the chamber volume in such devices leads to flow stagnation in the chamber and insufficient local perfusion near the cells.

Energy sources to drive the micropumps are also required. Biofuel cells (BFCs) are suitable energy sources for perfusion because they operate using biofuels such as glucose and oxygen. Other researchers have developed BFCs implanted in the body [21,22]. However, there are only few state-of-the-art implementations meeting the above requirements.

We have previously proposed a self-propelled tube-shaped microswimmer that integrates these BFC and EOF mechanisms [23]. Using standard UV photolithography, prototypes 100 μm in size were fabricated, and demonstrated a self-propulsion velocity of several 10 μm/s in glucose solution. We also successfully fabricated prototypes of 10 μm size using 3D laser lithography with two-photon absorption [24]. These demonstrated a self-propulsion velocity of several 100 μm/s, i.e., smaller and faster features than those prepared using UV photolithography. This microswimmers had only one through-hole generating EOF.

Using the principle of this microswimmer, we proposed a new concept of a film-shaped micropump array integrating BFC and EOF mechanisms [25]. Both end surfaces of the film-shaped micropump array generate open-circuit potential (OCP) as BFC in glucose solution. It has an array of multiple through-holes on the surface, each of which acts as a micropump to generate EOF due to the OCP. This could be a solution to the requirements of small size, wireless transmission, and size adjustability. Furthermore, this can be cut to any desired size according to the intended purpose. Therefore, the micropump array is expected to generate a flow in the environment when immersed in a fluid as shown in Figure 1b. This concept allows for local perfusion even in areas where flow tends to stagnate, and has the potential to efficiently culture all cell samples, regardless of their size.

This paper reports the detailed concept, design, and fabrication process of the film micropump array, and the evaluation of basic performance using fabricated prototypes. For basic performance, OCP and flow velocities generated in glucose solution were evaluated.

## 2. Materials and Methods

### 2.1. Concept

**Figure 2:**
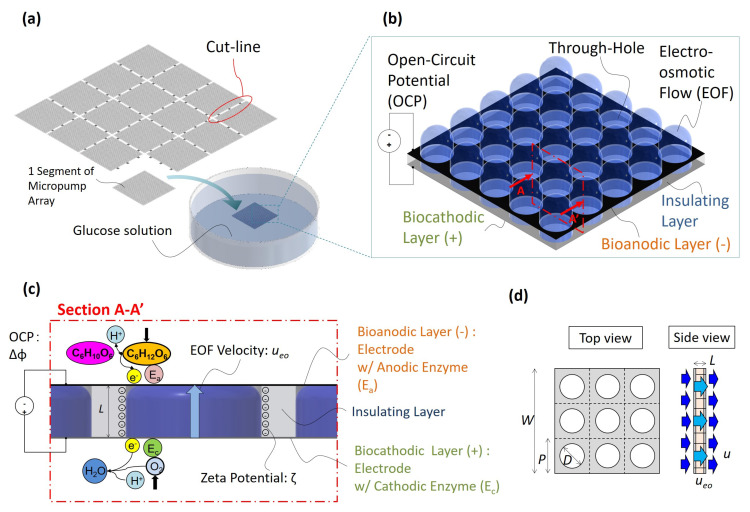
Concept of a film-shaped micropump array. (**a**) Size-adjustable configuration consisting of multiple segments and cut-lines. (**b**) Minimal configuration. (**c**) BFC and EOF mechanisms with related parameters. (**d**) Other design parameters.

As shown in Figure 2a, a film-shaped micropump array consists of multiple square segments. The side lengths of the segments are several mm. Two adjacent segments are connected by cut-lines consisting of multiple hinges. One or more segments can be easily cut along cut-lines, similar to postage stamps, and used according to the intended purpose. A segment is a film-like shape integrating a planar BFC and an EO micropump array, as shown in Figure 2b,c. It is composed of two electrode layers and one insulating layer located between them. It contains multiple through-holes, that penetrate in the direction of thickness, and are arranged in an array on the film surface. Two electrode layers, i.e., a bioanode layer and a biocathode layer, decompose glucose and oxygen in solution through an enzymatic redox reaction, and an OCP is generated between them. An EOF velocity $u_{eo}$ is then generated inside each through-hole depending on the OCP Δϕ from the electrode layers, the thickness *L* of the insulating layer, and the zeta potential ζ on the surface of the through-hole. Thus, the two electrode layers and the insulating layer function as a planar BFC and EO micropump array, respectively. Flow velocity *u* and flow rate *Q* outside the micropump array are also decided by the EOF velocity $u_{eo}$, the through-hole inner diameter *D*, the array pitch *P*, and the lateral length *W*.

### 2.2. Configuration and Design

Based on the standard UV photolithography used in previous research [23], we used the photoresist SU-8 as the material of the insulating layer, and conductive polymer composites (CPCs) composed of SU-8 and 7 vol% of enzyme-immobilized silver nanoparticles (AgNPs) as the material of the electrode layers. Glucose oxidase (GOx) was used as the anodic enzyme and laccase (LAC) as the cathodic enzyme.

Design parameters are presented in Table 1. The insulation layer thickness *L* was set to 30 μm as the thinnest size that can be handled without breaking. Each electrode layer is a few μm thick, much less than *L*. The diameter *D* and array pitch *P* of the through-holes were designed to be 70 μm and 100 μm, respectively. The *D* was determined considering the size that can be fabricated using photolithography. *P* was set close to *D* as long as the structure could be maintained without breaking.

### 2.3. Theoretical Model

The BFC mechanism consists of a bioanode with *GOx* and a biocathode with *LAC*, as shown in Figure 2c. On those electrodes, the redox reactions that occur are as follows:

(1) $$\begin{array}{ccc} \left(Bioanode\right) & : & C_{6}H_{12}O_{6}\overset{GOx}{\to }C_{6}H_{10}O_{6}+2H^{+}+2e^{-}, \end{array}$$

(2) $$\begin{array}{ccc} \left(Biocathode\right) & : & \frac{1}{2}O_{2}+2H^{+}+2e^{-}\overset{LAC}{\to }H_{2}O. \end{array}$$

By the above reactions, the OCP Δϕ is generated.

From microfluidic theory [26], the EOF velocity $u_{eo}$ inside each through-hole is described as follows:

(3) $$\begin{array}{ccc} u_{eo} & = & \frac{\epsilon \zeta }{\eta }\frac{\Delta \phi }{L}, \end{array}$$

where ϵ and η are the permittivity and viscosity of the glucose solution, respectively. From Equation (3), $u_{eo}$ is proportional to Δϕ and ζ, and inversely proportional to η and *L*.

As shown in Figure 2d, there is a flow conservation relationship between the planar region and all through-holes, so the mean planar flow velocity *u* in the vicinity of the micropump array is determined as follows:

(4) $$\begin{array}{ccc} u & = & \frac{\pi }{4}{\left(\frac{D}{P}\right)}^{2}u_{eo}=\frac{\pi }{4}{\left(\frac{D}{P}\right)}^{2}\frac{\epsilon \zeta }{\eta }\frac{\Delta \phi }{L}. \end{array}$$

The through-holes’ inner diameter *D* close to the array pitch *P* is suitable for efficiently converting EOFs to planar flow.

The mean flow rate *Q* is also described using the area $W^{2}$ of the micropump array as follows:

(5) $$\begin{array}{ccc} Q & = & W^{2}u=\frac{\pi }{4}{\left(\frac{WD}{P}\right)}^{2}u_{eo}=\frac{\pi }{4}{\left(\frac{WD}{P}\right)}^{2}\frac{\epsilon \zeta }{\eta }\frac{\Delta \phi }{L}. \end{array}$$

**Table 1:** Design parameters and estimated performance.

Meaning	Part	Symbol	Value (Units)	Comment
Permittivity	Glucose solution	ϵ	6.580 × 10${}^{-10}$ (F/m)	Water (310 K)
Viscosity	Glucose solution	η	0.692 × 10${}^{-3}$ (Pa s)	Water (310 K)
Mass density	Glucose solution	ρ	993 (kg/m${}^{3}$)	Water (310 K)
OCP	Electrode layers	Δϕ	≥100 (mV)	[23]
Zeta potential	Insulating layer	ζ	−30 (mV)	SU-8 [27]
Thickness	Insulating layer	*L*	30 (μm)	–
Inner diameter	Through-hole	*D*	70 (μm)	–
Array pitch	Through-hole	*P*	100 (μm)	–
Lateral size	Micropump array	*W*	3 (mm)	–
EOF velocity	Micropump array	$u_{eo}$	≥95 (μm/s)	–
Mean planar flow velocity	Micropump array	*u*	≥37 (μm/s)	–
Mean flow rate	Micropump array	*Q*	≥20 (μL/min)	–

### 2.4. Theoretical Performance

Theoretical supremum of the OCP in this configuration is estimated as 1.18 V [28] calculated using the Gibbs free energy of the reaction Equations (1) and (2). In real applications, the OCP achieved depends on the design, the manufacturing method, and the experimental conditions. Zebda et al. demonstrated an OCP of 0.95 V experimentally [29]. The OCP is generally highly dependent on the fuel concentration and the fuel flux to the electrodes. In our previous research [23], we simply checked the OCP before and after immersion of a different BFC test substrate in 67 mM glucose solution by using a digital multimeter. In that simple measurement, the OCP fluctuated, but at least 100 mV or more was measured. The same order of magnitude is also assumed for this configuration.

The theoretical EOF velocity $u_{eo}$ was estimated to be at least 95 μm/s using Equation (3) with the design parameter values shown in Table 1. In that case, the mean planar flow velocity *u* and mean flow rate *Q* were calculated as 37 μm/s and 20 μL/min, respectively, using Equations (4) and (5). Their performance is highly dependent on the OCP Δϕ of the BFC mechanism. Because of the Reynolds number Re =ρuW/η=0.16<1, it is expected that so-called Stokes flows [26], in which viscous forces dominate, will occur around the pump array.

**Figure 3:**
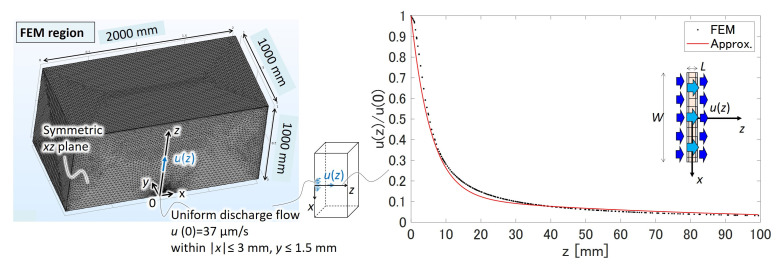
Velocity decay characteristic along the vertical direction (*z*) away from the pump array estimated using finite element method (FEM) analysis of laminar flow using COMSOL Multiphysics (COMSOL, Inc., Stockholm, Sweden). The lateral size of the pump array was 3 mm and u(0) was set as 37.0 μm/s. The approximation curve was identified as 0.875exp(−z/5.71 mm) + (1−0.875)exp(−z/81.3 mm). The approximation was performed using the Curve Fitting Toolbox in MATLAB (The MathWorks, Inc., Natick, MA, USA) with a coefficient of determination of 0.9833.

Figure 3 shows the velocity decay characteristic u(z) along the vertical direction *z* away from the pump array, estimated using finite element method (FEM) analysis of laminar flow using COMSOL Multiphysics (COMSOL, Inc, Stockholm, Sweden). The lateral size of the pump array was 3 mm and u(0) was set as 37.0 μm/s. In the analysis, a sufficiently large area of |x|≤ 1000 mm, |y|≤ 1000 mm, and z≤ 1000 mm around the pump array (*z* = 0) was considered and the parameters presented in Table 1 were used. Flows with u(z) > 0 at *z* > 0 mean discharge from the micropump array. Since these are Stokes flows, suction flows with u(z) > 0 occur to the same degree at *z* < 0. Therefore, the approximation curve of u(z) was identified as follows:

(6) $$\begin{array}{ccc} \frac{u(z)}{u(0)} & = & 0.875e^{-\frac{\left|z\right|}{5.71[mm]}}+\left(1-0.875\right)e^{-\frac{\left|z\right|}{81.3[mm]}}. \end{array}$$

The approximation was performed using the Curve Fitting Toolbox in MATLAB (The MathWorks, Inc., Natick, MA, USA) with a coefficient of determination of 0.9833. This calculation can be used to consider the placement of multiple micropump arrays in perfusion applications. For example, at a distance of 5 mm and 30 mm from the pump array, u(z) is expected to decrease to about 50% and 10% of u(0), respectively.

### 2.5. Fabrication Process

**Figure 4:**
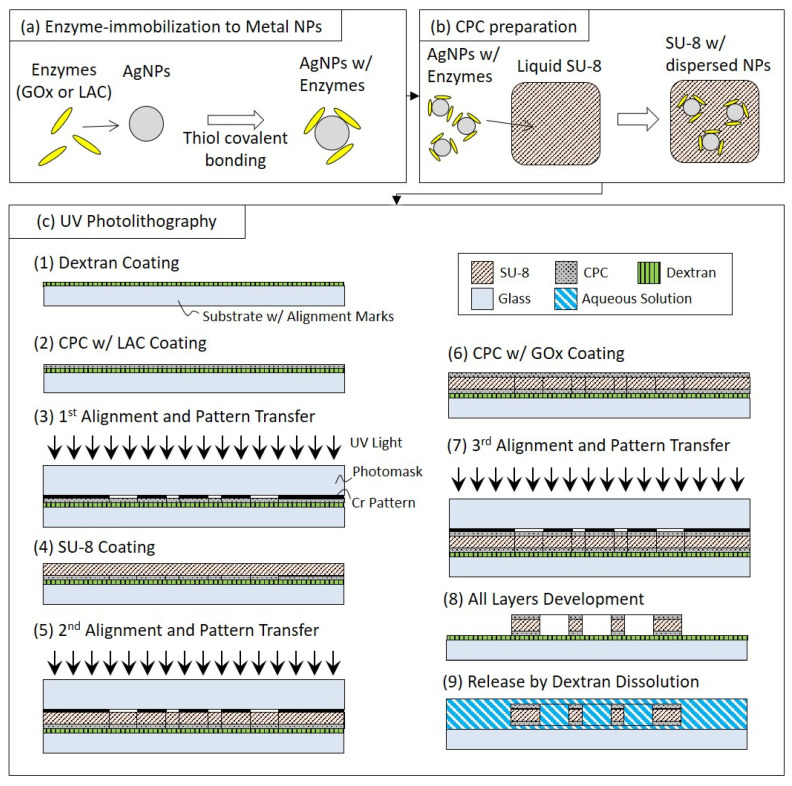
Fabrication process. (**a**) Enzyme-immobilization to metal NPs with a covalent bonding method using SAM of alkanethiols. (**b**) CPC preparation by dispersing the enzyme-immobilized metal NPs in uncured SU-8. (**c**) UV photolithography with mask pattern transfers.

Figure 4 shows the fabrication process. All materials not specifically mentioned were purchased from Sigma-Aldrich (Saint Louis, MO, USA).

#### 2.5.1. Enzyme-Immobilization to Metal NPs

First, enzyme-immobilization to AgNPs was performed (Figure 4a). Enzymes (GOx or LAC) should be immobilized on the surface of NPs to facilitate direct electron transfer. Many methods for immobilizing enzymes have been reported [30], and we applied the covalent bonding method of the enzymes to the metal using a self-assembled monolayer (SAM) of alkanethiols formed on the metal surface [31,32]. We used 11-mercaptoundecanoic acid (11-MUA) as the alkanethiol. To form SAMs, AgNPs (35 nm in diameter, IoLiTec GmbH) were immersed in 1100 mM 11-MUA solution in cyclopentanone for more than 300 min using a 1.2 mL microtube. Cyclopentanone is a suitable solvent because it is also the solvent for SU-8 3000 resists (KAYAKU Advanced Materials, Inc., Westborough, MA, USA) used in the subsequent process. N,N’-dicyclohexylcarbodiimide (DCC) and N-hydroxysuccinimide (NHS) were used to make the AgNP surfaces reactive [33]. Centrifuged AgNPs with 11-MUA SAMs were immersed in 200 mM DCC and 50 mM NHS solution in cyclopentanone for 30 min. To immobilize enzymes on the AgNPs’ surfaces, centrifuged AgNPs were immersed in 15 mg/mL GOx or 100 mg/mL LAC dispersion in cyclopentanone for at least 60 min. Finally, the enzyme-immobilized AgNPs were obtained by removing excess enzymes via centrifugation.

#### 2.5.2. CPC Preparation

The CPC was then prepared by dispersing the enzyme-immobilized AgNPs in uncured SU-8 (Figure 4b). The concentration of SU-8 resin in the solvent was adjusted to 25 vol% by adding cyclopentanone to SU-8 3005 (50 vol% resin, KAYAKU Advanced Materials, Inc., Westborough, MA, USA). This low concentration was used to obtain a thin film of about 1 μm thickness of SU-8 resin. Additionally, the concentration of AgNPs in the resin was prepared at 7 vol% because the concentration must be at least 6 vol% for the polymer composite to become conductive [34].

#### 2.5.3. UV Photolithography with Mask Pattern Transfers

Next, pattern transfer using standard UV photolithography was executed using a mask aligner (Suss MA6, SUSS MicroTech SE, Munich, Germany) (Figure 4c). A dextran layer (10 *w*/*v*% solution in deionized water) was spin-coated on a glass substrate at 2000 rpm for 45 s and baked at 150 ${}^{\circ }$C for 2 min. It served as a water-soluble sacrificial layer [35] with a thickness of about 0.3 μm. In all subsequent processes, the baking temperature was set to 65 ${}^{\circ }$C to minimize enzyme inactivation [36]. Prepared CPC with LAC was spin-coated on top of the sacrificial layer at 5000 rpm for 60 s and baked for more than 10 min to obtain a biocathodic layer with 1 μm thickness. The patterns of the prototype design were transferred to the layer using UV exposure with more than 3000 mJ/cm${}^{2}$ after alignment. Such a relatively large illumination is required to polymerize CPC because of its opacity. After postbaking for 5 min, SU-8 3025 (KAYAKU Advanced Materials, Inc., Westborough, MA, USA) was spin-coated on top at 2550 rpm for 60 s and baked over 60 min to obtain a 30 μm thick insulating layer. The same patterns were transferred to the insulating layer by exposure to UV with 100 mJ/cm${}^{2}$ after alignment. After postbaking for 5 min, spin-coating of a bioanodic layer composed of CPC with GOx, and pattern transfer onto it were performed under the same conditions as for the biocathodic layer. After postbaking for 5 min, the patterns were chemically developed using PM thinner (Tokyo Ohka Kogyo Co., Ltd., Kawasaki, Japan) over 10 min and gently rinsed with isopropyl alcohol for 1 min. Finally, the substrate was immersed in deionized water for about 1 min to dissolve the dextran layer and the finished prototypes were obtained.

### 2.6. Fabricated Prototype

**Figure 5:**
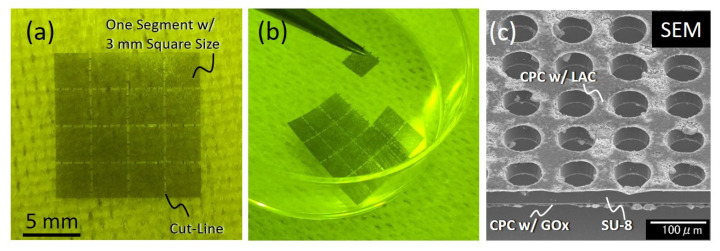
Fabricated prototypes of the film-shaped micropump array. (**a**) Overview of 4 × 4 segments with 3 mm square size. (**b**) One scene where a segment is separated along a cut line like a postage stamp. (**c**) Scanning electron microscope (SEM) image of the area around the through-hole array.

Figure 5 shows fabricated prototypes of the film-shaped micropump array. Film-shaped prototypes consisting of 4 by 4 segments connected by cut-lines were successfully fabricated (Figure 5a). Like a postage stamp, one segment with the square size of 3 mm can be easily cut along cut-lines and handled with tweezers (Figure 5b). Figure 5c shows a scanning electron microscope (SEM) image of the area around the through-hole array. The thicknesses of the insulating layer and each electrode layer were 30 μm and 1 μm, respectively. The diameter and array pitch of through-holes were 70 μm and 100 μm, respectively. Thus, prototypes were confirmed to be as designed.

### 2.7. Experimental Methods

**Figure 6:**
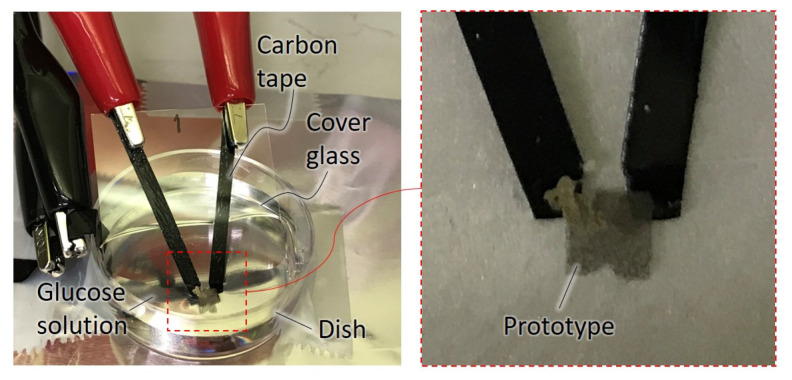
Experimental setup to measure OCP of a prototype in glucose solution.

We used an experimental setup to measure and evaluate OCP Δϕ of the prototypes in glucose solution as shown in Figure 6. A prototype was fixed to a cover glass with a carbon tape on each end face. Two carbon tapes were connected to an oscilloscope. The OCP between both end faces could be measured by immersing the cover glass in a glucose solution.

**Figure 7:**
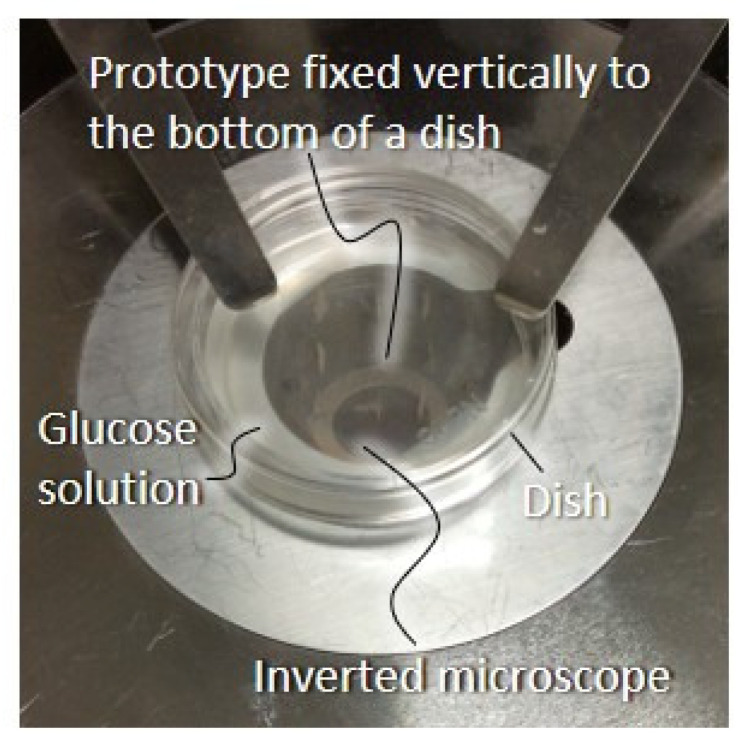
Experimental setup to observe flows around a prototype in glucose solution (67 mM β-D-glucose, 150 mM NaCl, and polystyrene (PS) beads with the size of 3 and 7 μm in 10 mM phosphate buffered saline, pH 7.4). The prototype was adhesively fixed vertically to the bottom of a dish.

Additionally, we adhesively fixed a prototype vertically on a small dish and observed flows in the vicinity perpendicular to the prototype in glucose solution (67 mM β-D-glucose, 150 mM NaCl, and polystyrene (PS) beads with the size of 3 and 7 μm in 10 mM phosphate buffered saline, pH 7.4) using an inverted microscope. Figure 7 shows the experimental setup. All time-lapse movies were acquired from the microscope at a sampling interval of 0.1 s for 30 s, after the dish had been left to rest for at least 30 min to eliminate other convections. After the acquisition of the movies, optical flow processing was performed using “OpenCV”. Blue points and white curves represent tracked particles such as the PS beads and their trajectories, and those indicated the surrounding flows. From the trajectories, the velocities perpendicular to the prototype plane can be identified as the generated planar flow velocities *u*.

## 3. Results and Discussion

### 3.1. OCP Generated by the Prototypes

**Figure 8:**
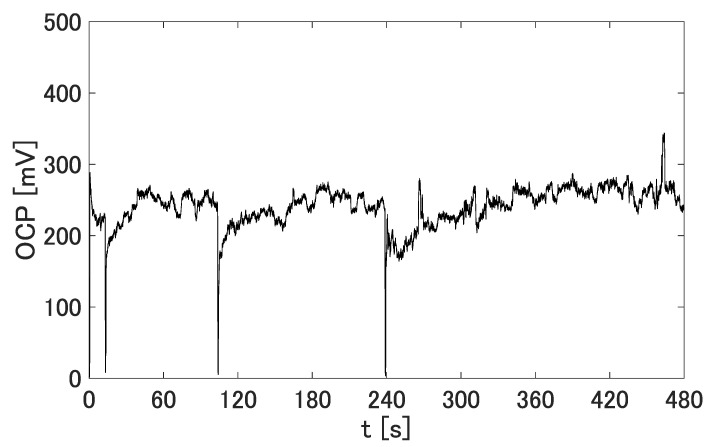
OCP measured after immersing a prototype in 8 mM glucose solution at t = 0. The sampling time was 10 ms and moving mean processing was performed for 10 points. Although OCP was relatively unstable, OCP with a maximum of 344 mV and a mean of 231 mV was observed for 480 s.

**Figure 9:**
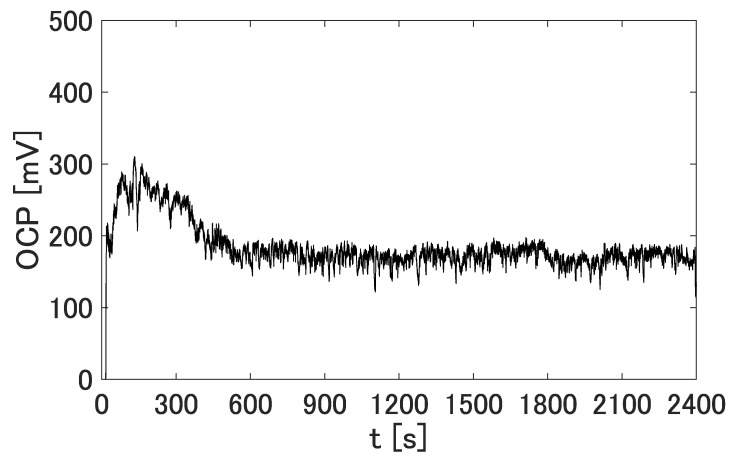
OCP measured after immersing a prototype in 67 mM glucose solution at t = 0. The sampling time was 10 ms and moving mean processing was performed for 10 points. A stable OCP was confirmed for at least 2000 s. A maximum OCP of 310 mV and a steady-state mean of 163 mV were observed.

Figure 8 shows the OCP measured after immersing a prototype in 8mM glucose solution at t = 0 using the setup as shown in Figure 6. The OCP was not always stable, at some moments of falling to zero, but a maximum OCP of 344 mV and a mean OCP of 231 mV were observed for 480 s. Figure 9 shows the OCP measured after immersing another prototype in 67 mM glucose solution at t = 0. In this case, a stable OCP was confirmed for at least 2000 s. A maximum OCP of 310 mV and a steady-state mean OCP of 163 mV were confirmed. For a relatively stable micropump array, it was confirmed that an OCP of about 200 mV could be acquired.

In the experiments, the measured OCPs and their occasional instability did not depend on the glucose concentration. The measured OCP is highly dependent on enzymes and deactivation of the enzymes may have occurred in any fabrication process. Control of parameters related to enzyme activity, such as temperature and pH, needs to be further improved throughout the total process. The cause of the instability is currently unknown, but it might be due to the electric condition of each through-hole in the prototype. For example, if one through-hole is electrically shorted due to attached impurities, the entire micropump array will also be electrically shorted. The OCP stability is expected to be enhanced in the future if the manufacturing stability of the electrode layers is improved and unnecessary AgNPs are completely removed. An electrode layer design that comprises multiple regions of equal potential in the plane is also considered to make the OCP more stable.

### 3.2. Generated Flows around the Prototypes

**Figure 10:**
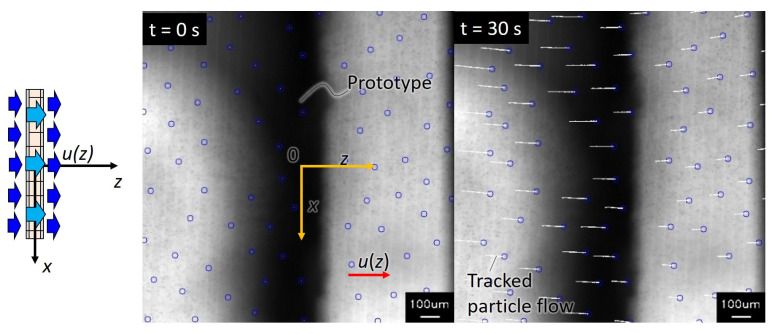
Snapshots from an observed video using optical-flow processing in “OpenCV”. The blue points and white curves represent tracked particles and their trajectories, respectively, and their mean flows.

**Figure 11:**
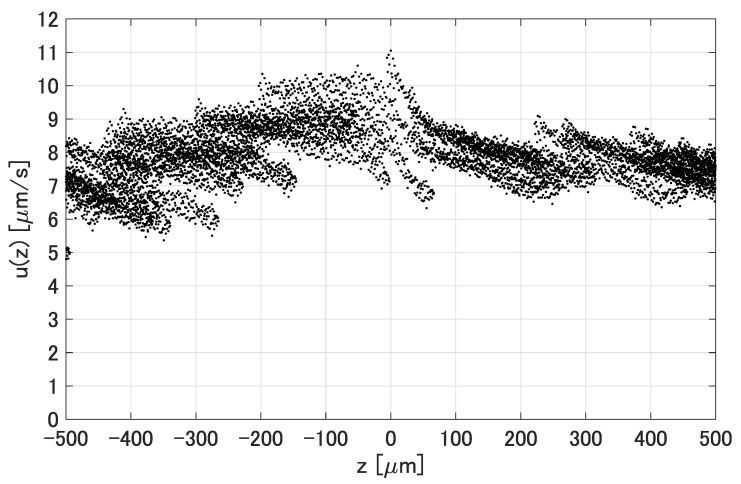
Flow velocity distribution near a prototype.

Flows around a prototype in the glucose solution were observed using an inverted microscope as shown in Figure 7. Figure 10 shows snapshots from an example of observed videos with optical-flow processing. Almost uniform flows in the direction perpendicular to the prototype plane were observed. Figure 11 shows an example of the spatial distribution of flow velocity *u* along the direction *z*. The *z* axis is perpendicular to the prototype plane and its origin represents the position on the center of the thickness. Flow velocities u(z) in the range of |z|≤ 500 μm for 30 s are plotted. As theoretically expected from the velocity decay characteristics depicted in Figure 3, *u* had a maximum value of 11.1 μm/s at the origin (*z* = 0) and a velocity decay was observed at |z| = 500 μm. The distribution of *u* shows almost symmetrical characteristics at the origin, implying that discharge (*z* > 0) and suction (*z* < 0) occurred at approximately the same degree across the pump position (*z* = 0). As noted above, these are considered Stokes flows, so this symmetrical distribution is reasonable. Because of the spatial distribution characteristics, it is considered that the flows were generated by the prototype.

**Figure 12:**
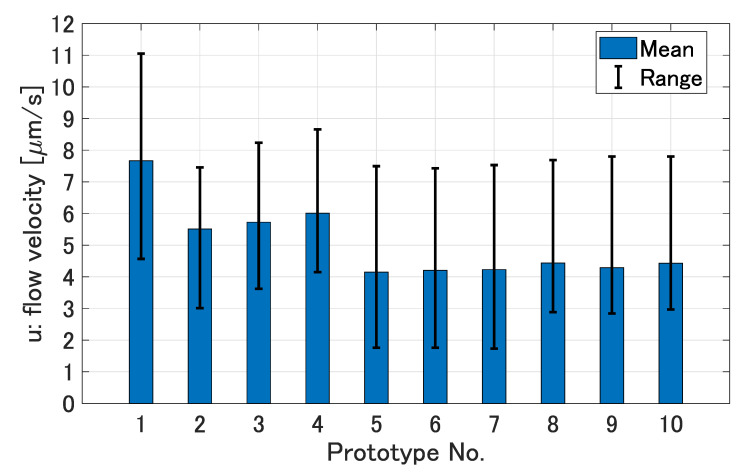
Flow velocity u(z) results of ten prototypes in the range of |z|≤ 500 μm for 30 s. Each prototype was observed for 30 s after left to rest for more than 30 min.

In all measurements depicted in Figure 11, the range and mean of flow velocities *u* were 7.9–11.1 μm/s and 9.1 μm/s, respectively, at *z* = 0, and were 4.8–8.4 μm/s and 7.2 μm/s, respectively, at |z| = 500 μm. Figure 12 summarizes u(z) results of ten prototypes in the range of |z|≤ 500 μm for 30 s. Thus this concept of micropump array was demonstrated.

However, theoretical flow velocity was expected as more than 37 μm/s as shown in Table 1. The reason for its performance difference is not exactly clear. As shown in the OCP results in Section 3.1, OCP was generated, although sometimes unstable. So, the main reason is assumed to be a reduction in the zeta potential. These factors need to be improved in future works.

## 4. Conclusions

In this paper we propose a new concept for a film-shaped micropump array integrating BFC and EOF mechanisms for biomedical use. We derived a flow velocity model and finalized the design based on performance estimates from that model. We fabricated the prototypes of the film-shaped micropump array using standard UV photolithography. The prototypes were small, thin, and wireless. Like postage stamps, the micropump array could be cut along cut-lines to the desired size according to the purpose. We demonstrated the OCP and flow generation of the prototypes in glucose solutions. A higher flow velocity and stability are the next issues. In the future, this concept is expected to be applied to perfusion of biological fluids at small local sites near or inside cultured cells, cultured tissues, living bodies, and so on.

## Abbreviations

The following abbreviations are used in this manuscript:

11-MUA	11-mercaptoundecanoic acid
AgNP	Silver nanoparticle
BFC	Biofuel cell
CPC	Conductive polymer composite
DCC	N,N’-dicyclohexylcarbodiimide
EO	Electro-osmotic
EOF	Electro-osmotic flow
GOx	Glucose oxidase
LAC	Laccase
NHS	N-hydroxysuccinimide
NP	Nanoparticle
OCP	Open-circuit potential
SAM	Self-assembled monolayer
SEM	Scanning electron microscope
UV	Ultraviolet
